# Risk Factors for Healthcare-Associated Extensively Drug-Resistant *Acinetobacter baumannii* Infections: A Case-Control Study

**DOI:** 10.1371/journal.pone.0085973

**Published:** 2014-01-21

**Authors:** Ming-Chin Chan, Sheng-Kang Chiu, Po-Ren Hsueh, Ning-Chi Wang, Chih-Chien Wang, Chi-Tai Fang

**Affiliations:** 1 Infection Control Office, Tri-Service General Hospital, National Defense Medical Center, Taipei, Taiwan; 2 Department of Internal Medicine, Tri-Service General Hospital, National Defense Medical Center, Taipei, Taiwan; 3 Department of Internal Medicine, National Taiwan University Hospital, National Taiwan University College of Medicine, Taipei, Taiwan; 4 Department of Laboratory Medicine, National Taiwan University Hospital, National Taiwan University College of Medicine, Taipei, Taiwan; 5 Department of Pediatrics, Tri-Service General Hospital, National Defense Medical Center, Taipei, Taiwan; 6 Institute of Epidemiology and Preventive Medicine, College of Public Health, National Taiwan University, Taipei, Taiwan; University of Iowa Carver College of Medicine, United States of America

## Abstract

The emergence of extensively drug-resistant *Acinetobacter baumannii* (XDRAB) is a serious threat to hospitalized patients. From 2008 to 2010, surveillance detected 25 hospital-acquired infection (HAI) cases caused by XDRAB at a medical center in Taipei. The site of XDRAB infection was bloodstream (n = 8), urinary tract (n = 12), lower respiratory tract (n = 3), surgical site (n = 1), and cardiovascular (n = 1). The isolates were resistant to all currently available antibiotics except for colistin. The XDRAB isolates are genetically diverse, shown by pulsed-field gel electrophoresis, but 23 of 25 harbored class 1 integron with a 2.3-kb gene cassette. Most of these isolates carry OXA-23 (n = 21) and OXA-51-like carbapenemase genes (n = 25). To identify the risk factors, a case-control study was conducted. The 25 cases were compared with 100 controls randomly selected from hospitalized patients without XDRAB-HAIs, matched by the onset date, ward, and age, at a ratio of 1∶4. Prior use of imipenem, meropenem, piperacillin/tazobactam or fourth-generation cephalosporins (adjusted OR: 3.2, 95% CI: 1.03–10.2, *P* = 0.04) and >30 days bed-ridden (adjusted OR: 6.0, 95% CI: 1.3–27.6, *P* = 0.02) were found to be the independent risk factors for XDRAB-HAIs. These findings highlight that, even in the absence of clonal dissemination, XDRAB can emerge under the selective pressure of broad-spectrum antibiotics and causes subsequent HAIs in compromised hosts. An appropriate response to the XDRAB threat therefore should include a component of prudent use of broad-spectrum antibiotics active against gram-negative bacteria.

## Introduction

*Acinetobacter baumannii* is an important pathogen that causes hospital-acquired infections (HAIs) worldwide [1], [2]. *A. baumannii* is characterized by its rapid development of drug resistance to all *β*-lactam antibiotics, indicating a high adaptability to the selective pressure from the extensive use of antibiotics [2]. Resistance to carbapenems in *A. baumannii* is mediated by the production of the carbapenemases OXA-23, OXA-24, OXA-58, and OXA-51 [3]–[5]. In addition, integrons that confer horizontal transfer of drug resistance gene cassettes have been reported in multidrug-resistant (MDR) *A. baumannii* [6]–[10]. HAIs caused by MDR *A. baumannii* were associated with a longer hospitalization and a higher mortality rate than that caused by non-MDR *A. baumannii* [1], [11]. The emergence of extensively drug-resistant *A. baumannii* (XDRAB) further limits the treatment options and constitutes a serious threat to HAI control. The prevalence of XDRAB among clinical *A. baumannii* isolates has reached 15% by 2005 and up to 41% by 2010 in Taiwan [12], [13], with evidences for clonal dissemination between hospitals [12], [14]. Such trends highlight an evolving challenge posed by XDRAB, and an urgent need for effective control and prevention measures.

From 2008 to 2010, surveillance at a medical center in Taipei detected 25 HAIs cases caused by XDRAB isolates resistant to all currently available antibiotics except for colistin. This study aimed to analyze the genetic linkage and drug-resistance gene profiles of these XDRAB isolates, and to identify the risk factors for XDRAB-HAIs using a matched case-control study.

## Methods

### Study Setting

This investigation was conducted at Tri-Service General Hospital (TSGH) (Taipei, Taiwan) from November 2008 to December 2010. TSGH is an 1800-bed medical center that provides both primary and tertiary referral care. The study procedures were reviewed and approved by the institutional review board of TSGH (protocol no. 100-05-159) as an HAI surveillance study, which does not require informed consent.

### HAI Surveillance

Infection control nurses routinely survey HAIs using the Centers for Disease Control and Prevention (CDC) HAI surveillance definitions [15]. Cases of XDRAB-HAI were retrospectively identified from HAI surveillance databases.

### Case Definition

XDRAB was defined as *A. baumannii* isolates resistant to all tested currently available antibiotics except for colistin by disk susceptibility test [16]. To be included as a case, the patient must have had an HAI, as defined by the CDC surveillance criteria [15], for which XDRAB must be the causal microorganism.

### Bacterial Isolates and Antibiotic Susceptibility Testing

Since November 2008, all clinical XDRAB isolates were routinely collected and stored at −80°C until use. Disk diffusion method was routinely used for susceptibility testing of *A. baumannii* isolates [16]. For tigecycline, a 15 µg disk with breakpoints of ≥16 mm (susceptible) and ≤12 mm (resistant) was used [17]. The minimum inhibitory concentrations (MICs) of tigecycline were also determined using E-test (AB Biodisk, Solna, Sweden). The British Society for Antimicrobial Chemotherapy (BSAC) tigecycline breakpoints (for *Acinetobacter* spp.: ≤1 µg/mL, susceptible; 2 µg/mL, intermediate; and >2 µg/mL, resistant) were used as the MIC interpretative criteria [18]. Strains collected from the same patient, regardless of the type of clinical specimen, were counted as one sample.

### Pulsed-Field Gel Electrophoresis

Pulsed-field gel electrophoresis (PFGE) was performed using previously described methods [19], [20]. In brief, the purified bacterial genomic DNA was digested by the restriction enzyme *Apa*I, and the fragments were separated in a CHEF Mapper system (Bio-Rad Laboratories, Hercules, CA, USA). The PFGE profiles were interpreted according to Tenover et al [20]. We use BioNumerics software (Applied Maths, Kortrijk, Belgium) to analyze similarities between digitized PFGE outputs. The Jaccard complete linkage method was used to analyze gene clustering.

### Integron Detection and OXA Typing

The detection of class 1 and class 2 integrases and the integron gene cassette and the typing of OXA genes were performed using PCR as previously described [21]–[23]. The primers are listed in Table S1. The result of OXA typing was further confirmed by nucleotide sequencing.

### Case-Control Study

For each XDRAB HAI case, four control inpatients without XDRAB-HAI were selected from hospitalized patients matched by onset date, ward, and age (within a 5-year range), using random sampling without replacement from the hospital admission registry. Clinical information on the XDRAB-HAI patients and matched controls, including underlying diseases, invasive medical procedures when XDRAB-HAI occurred, and the prior use of antibiotics during the time period from the most recent admission date to the XDRAB-HAI onset date (for those with prolonged hospitalization, only antibiotic use within 3 months before the XDRAB-HAI onset was counted), were collected systematically from medical records using a computerized data form.

### Statistical Analysis

The clinical data collected from the XDRAB-HAI and matched control patients were analyzed using conditional logistic regression. Variables reached statistical significance in the univariate analyses were further included in the multivariate analyses. All analyses were performed using SAS software version 9.2 (SAS Institute, Cary, North Carolina, USA). P values <0.05 are considered statistically significant.

## Results

### Characteristics of XDRAB-HAI Cases

The 25 XDRAB-HAI cases occurred in 10 patients at intensive care units and 15 patients at the general ward, respectively; 18 of them were male, and 7 were female; 21 of the 25 patients were older than 65 years. The XDRAB infection sites included bloodstream infection (n = 8), urinary tract infection (n = 12), lower respiratory tract infection (n = 3), surgical site infection (n = 1), and cardiovascular infection (n = 1).

### Antimicrobial Susceptibility of XDRAB Isolates

The 25 XDRAB isolates were resistant to all cephalosporins, all aminoglycosides, imipenem, ciprofloxacin, ampicillin/sulbactam, piperacillin/tazobactam, sulfamethoxazole-trimethoprim, and tigecycline by disk diffusion method. The tigecycline MICs were 16 µg/mL in 4 isolates, 8 µg/mL in 10 isolates, 4 µg/mL in 6 isolates, 2 µg/mL in 4 isolates, and 0.5 µg/mL in 1 isolate (MIC_50_: 8 µg/mL; MIC_90_: 16 µg/mL). Using the BSAC interpretive criteria, 24 of the 25 isolates were not susceptible to tigecycline.

### PFGE Typing of XDRAB Isolates

**Figure 1:**
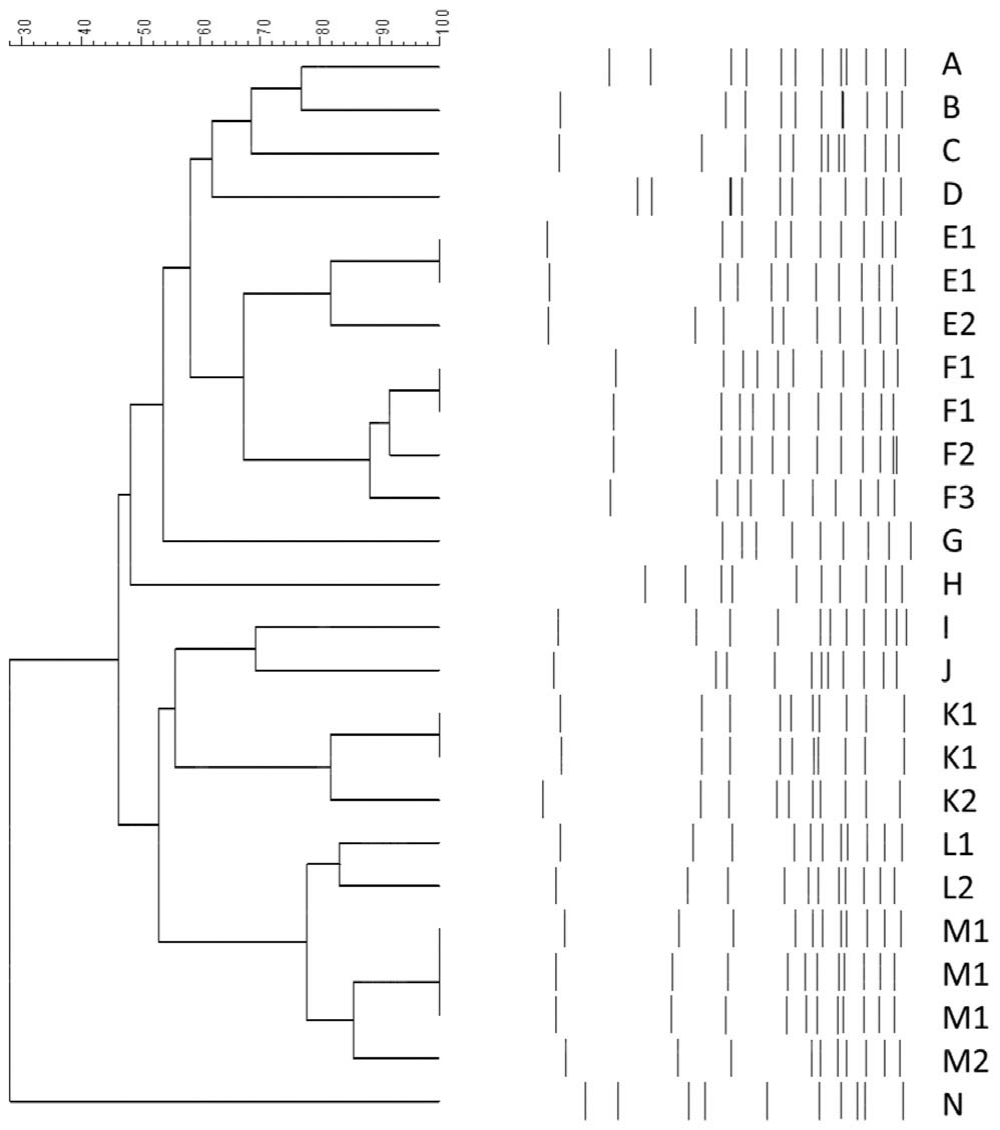
Pulsed-field gel electrophoresis (PFGE) dendrogram of XRDAB. The scale indicates the percentage of genetic similarity. 14 pulsotypes (A–N) and 20 pulsosubtypes (A–D, E1–E2, F1–F3, G–J, K1–K2, L1–L2, M1–M2, N) were identified.

Figure 1 shows the PFGE results of the XDRAB isolates collected from the 25 infected patients, and the results are presented as a dendrogram. Figure 1 shows the XDRAB isolates recovered from the 25 infected patients typed into 14 pulsotypes (the isolates with >80% similarity were considered to belong to the same pulsotype) and 20 pulsosubtypes. There was no single predominant pulsotype.

Further investigation revealed that the isolates were collected from the following areas: 2 pulsotype E1 isolates were collected from the respiratory care center and the surgical intensive care unit (the collection dates were November, 2008 and March, 2009, respectively); the two pulsotype F1 isolates were obtained from Ward 31 and Ward 32, respectively (the collection dates were November, 2008 and June, 2009, respectively); 2 pulsotype K1 isolates were collected from Ward 51 and the cardiology intensive care unit, respectively (the collection dates were September, 2010 and November, 2010, respectively); and the 3 pulsotype M1 isolates were recovered from the medical intensive care unit, Ward 15, and Ward 31, respectively (the collection dates were December, 2009, June, 2009, and July, 2009, respectively). No significant correlation between the date of collection and the rooms of isolates with identical pulsotypes was found.

### Class 1 integron and OXA Carbapenemase Genes in XDRAB Isolates

Twenty-three (92%) of the 25 XDRAB isolates carried class 1 integrase and a 2.3-kb integron gene cassette (Table 1). No class 2 integron was detected. The results of OXA typing further revealed that most isolates carried either OXA-23 (21 isolates, 84%) or OXA-51-like carbapenemase genes (25 isolates, 100%). The 23 XDRAB isolates that carried class 1 integron all carried OXA-51-like carbapenemase gene. Meanwhile, 19 isolates carried both OXA-23 and OXA-51-like carbapenemase genes, and 1 of the 19 isolates carried OXA-24 and OXA-51-like carbapenemase genes. The 2 isolates that did not carry either class 1 or class 2 integron both carried OXA-23 and OXA-51-like carbapenemase genes (Table 1).

**Table 1:** Integron profiles and OXA molecular typing results of XDRAB isolates.

Number	Case number	Clone number	Class 1 Integrase	Class 2 Integrase	Integron gene cassette (2300 bp)	OXA-24 (246 bp)	OXA-51 (353 bp)	OXA-23 (501 bp)	OXA-58 (599 bp)
1	33	90	+	−	+	−	+	−	−
2	34	96	+	−	+	−	+	+	−
3	35	92	+	−	+	−	+	+	−
4	36	98	−	−	−	−	+	+	−
5	37	134	+	−	+	−	+	+	−
6	38	169	+	−	+	−	+	+	−
7	39	158	+	−	+	−	+	+	−
8	40	173	+	−	+	−	+	+	−
9	41	182	+	−	+	−	+	+	−
10	43	179	+	−	+	−	+	+	−
11	44	205	+	−	+	−	+	-	−
12	45	201	+	−	+	−	+	+	−
13	46	210	+	−	+	−	+	+	−
14	47	213	+	−	+	−	+	+	−
15	49	250	+	−	+	−	+	+	−
16	51	269	−	−	−	−	+	+	−
17	53	299	+	−	+	−	+	+	−
18	54	315	+	−	+	−	+	+	−
19	55	272	+	−	+	−	+	−	−
20	56	340	+	−	+	−	+	+	−
21	57	343	+	−	+	−	+	+	−
22	58	353	+	−	+	−	+	+	−
23	89	342	+	−	+	−	+	+	−
24	60	357	+	−	+	−	+	+	−
25	61	364	+	−	+	+	+	−	−

**Note**: “+” means PCR products were detected. “−” means PCR products were not detected.

### Risk Factors for XDRAB-HAIs

Univariate conditional logistic regression analyses of data from the 25 XDRAB-HAI cases and 100 individually matched controls showed that >30 days bed-ridden, hemodialysis using a PermCath™ or dual lumen catheter, tracheotomy, and prior use of glycopeptides, imipenem or meropenem, piperacillin/tazobactam, and fourth-generation cephalosporins were significant risk factors (Table 2). In the multivariate analysis, >30 days bed-ridden (adjusted OR: 6.0, 95% CI: 1.3–27.6, *P* = 0.02) and prior use of imipenem, meropenem, piperacillin/tazobactam or fourth-generation cephalosporins (adjusted OR: 3.2, 95% CI: 1.03–10.2, *P* = 0.04) were independent risk factors for XDRAB-HAIs (Table 3).

**Table 2:** Risk factors for XDRAB-HAIs: univariate analyses.

	Case		Control		Univariate logistic regression		
Variables	Yes	No	Yes	No	OR	(95% CI)	P value
Age ≥65 years	21	4	84	16	0.8	(0.7–1.0)	0.09
Sex: Male/Female	18	7	51	49	2.4	(1.0–6.0)	0.07
Underlying disease							
Solid tumor	8	17	24	76	1.5	(0.6–4.0)	0.41
Hematologic malignancy	0	25	1	99	-		
CVA	3	22	29	71	0.4	(0.1–1.2)	0.10
DM	6	19	35	65	0.6	(0.2–1.6)	0.29
Uremia	3	22	3	97	4.0	(0.8–19.8)	0.09
Liver cirrhosis	2	23	4	96	2.0	(0.4–10.9)	0.42
SLE	1	24	2	98	2.5	(0.1–42.6)	0.54
Implant	1	24	9	91	0.4	(0.1–3.5)	0.43
>30 days bed-ridden	8	17	6	94	7.0	(2.1–23.5)	<0.01*
Steroid usage	2	23	6	94	1.4	(0.3–7.2)	0.72
Chemotherapy/Radiotherapy	0	25	4	96	-		
Coma	0	25	1	99	-		
Invasive procedure							
Peripheral IV	17	8	81	19	0.4	(0.2–1.3)	0.13
CVP	10	15	34	66	1.4	(0.5–3.6)	0.55
Long-term IV	0	25	2	98	-		
CPN	5	20	10	90	2.4	(0.7–8.2)	0.17
Arterial line	5	20	27	73	0.5	(0.1–2.0)	0.31
Swan-Ganz	1	24	4	96	1.0	(0.1–10.1)	1.00
HD (A–V fistula/graft)	2	23	3	97	2.7	(0.5–16.0)	0.28
HD (Perm/Double lumen)	5	20	6	94	3.7	(1.01–12.8)	0.04*
Foley catheter	14	11	45	55	1.9	(0.7–5.3)	0.25
Endotracheal	5	20	25	75	0.7	(0.3–2.2)	0.60
Tracheostomy	13	12	27	73	3.2	(1.2–8.2)	0.02*
Respirator	11	14	38	62	1.3	(0.5–3.1)	0.58
Drainage catheter	1	24	22	78	0.2	(0.0–1.2)	0.07
Prior antibiotic usage							
Glycopeptide	7	18	11	89	3.6	(1.1–11.9)	0.03*
Aminoglycoside	3	22	15	85	0.7	(0.2–3.2)	0.67
Ertapenem	3	22	7	93	1.8	(0.4–7.2)	0.42
Imipenem or meropenem	5	20	7	93	4.5	(1.01–19.7)	0.049*
Tigecycline	1	24	5	95	0.8	(0.1–8.5)	0.81
Anti-Pseudomonal penicillins	13	12	19	81	5.1	(1.9–14.0)	<0.01*
3^rd^ Cephalosporin	8	17	23	77	1.5	(0.6–3.9)	0.37
4^th^ Cephalosporin	10	15	18	82	2.7	(1.1–6.8)	0.03*
Anti-pseudomonal cephalosporins	10	15	28	72	1.7	(0.7–4.3)	0.25
Quinolone	11	14	40	60	1.2	(0.4–2.8)	0.72

**Note**: Data are numbers of patients, unless otherwise indicated.

OR: odds ratio.

*Statistically significant.

CVA: Cerebral vascular accident. DM: Diabetes mellitus. CVP: Central venous pressure catheter. CPN: Central parenteral nutrition. HD: Hemodialysis.

Anti- Pseudomonal penicillins: piperacillin/tazobactam.

Anti-Pseudomonal cephalosporins: cefepime, cefpirome and ceftazidime.

**Table 3:** Risk factors for XDRAB-HAIs: multivariate analyses.

	Case (N = 25)		Controls (N = 100)		Univariate Logistic regression			Multivariate Logistic regression		
Variables	Yes	No	Yes	No	OR	(95% CI)	P value	OR	(95% CI)	P value
>30 days bed-ridden	8	17	6	94	7.0	(2.1–23.5)	<0.01	6.0	(1.3–27.6)	0.02*
HD (Perm/Double lumen)	5	20	6	94	3.7	(1.01–12.8)	0.04	1.9	(0.4–8.4)	0.40
Tracheostomy	13	12	27	73	3.2	(1.2–8.2)	0.02	1.8	(0.6–5.4)	0.29
Glycopeptide	7	18	11	89	3.6	(1.1–11.9)	0.03	1.5	(0.4–5.5)	0.55
Imipenem, meropenem, anti-pseudomonal penicillins, or 4th gen Cephalosporin usage	18	7	37	63	4.3	(1.6–11.4)	<0.01	3.2	(1.03–10.2)	0.04*

**Note**: Data are numbers of patients, unless otherwise indicated.

OR: odds ratio.

*Statistically significant.

## Discussion

Unlike the two previous reports on the clonal dissemination of XDRAB within a hospital [24] or between hospitals [12], [13] in Taiwan, the 25 XDRAB isolates in this investigation were genetically diverse, as demonstrated by PFGE. On the other hand, prior use of imipenem or meropenem, piperacillin/tazobactam, or fourth-generation cephalosporins were found to be significant risk factors for XDRAB-HAIs. Therefore, in addition to screening, isolation, and disinfection to contain clonal dissemination, reducing selection pressure that drives microbial adaptation by prudent use of broad-spectrum antibiotics against gram-negative bacteria is also essential for the prevention of XDRAB-HAIs.

The mechanism of extensively drug resistance in *A. baumannii* has not yet been fully understood. Multiple mechanisms are likely to work in synergism to produce this phenotype. All of the 25 XDRAB isolates carried OXA-51-like carbapenemase gene (100%), confirming the previous reports that OXA-51 is naturally present on the *A. baumannii* chromosome [25], [26]. Twenty-one of the 25 isolates also carried OXA-23 (84%), a finding consistent with previous observations in Taiwan [27], Korea [28] and China [29] that OXA-23 is commonly present in carbapenem-resistant *A. baumannii* isolates. Previous studies reported that integrons could play an important role in transfer of resistance to aminoglycosides, carbapenem, and chloramphenicol [6]–[10]. In the present study, we found that 23 (92%) of the 25 XDRAB isolates carried class 1 integron with a 2.3-kb gene cassette. The presence of class 1 integron in genetically diverse XDRAB isolates suggested that drug resistance genes contribute to the extensive drug resistance phenotype might be horizontally transferred between different *A. baumannii* strains.

The identification of XDRAB-HAI cases in this investigation was based on the routine disk susceptibility testing performed by the clinical microbiology laboratory in the hospital. We further performed MIC testing to confirm the nonsusceptibility to tigecycline in the 25 XDRAB isolates, and found that the MIC ranged from 0.5 to 16 µg/mL, with an MIC_50_ of 8 µg/mL and an MIC_90_ of 16 µg/mL. Both Clinical and Laboratory Standards Institute (CLSI) and the European Committee on Antimicrobial Susceptibility Testing (EUCAST) have not yet provided a reference for tigecycline MIC breakpoints. Using BSAC tigecycline breakpoints for *Acinetobacter* spp. [18] as interpretive criteria, 24 (96%) of the 25 isolates were not susceptible to tigecycline. Compared with the tigecycline MICs reported in a previous XDRAB investigation in South Korea (range: 1–8 mg/L, MIC_50_: 2 mg/L; MIC_90_: 4 mg/L) [28], the tigecycline MICs of the 25 XDRAB isolates in the present investigation were significantly higher (MIC_50_: 8 µg/mL; MIC_90_: 16 µg/mL), and colistin would be the only remaining treatment option.

The severity of underlying diseases, as measured by the Acute Physiology and Chronic Health Evaluation (APACHE) II score, has been found to be an independent risk factor for XDRAB acquisition [28]. Using hospitalized patients without an XDRAB-HAI as the controls, matched by onset date, age, and ward, we identified several host risk factors for XDRAB-HAIs, including >30 days bed-ridden, tracheotomy, and hemodialysis with catheter placement; >30 days bed-ridden was identified as an independent host risk factor. Our results indicate that compromised hosts are at increased risk for XDRAB-HAIs.

We found that broad-spectrum antibiotics with activity against gram-negative bacteria, including imipenem/meropenem, piperacillin/tazobactam, or 4th-generation cephalosporins, were significant risk factors for subsequent XDRAB-HAIs in both the univariate and multivariate analyses (Table 2 and Table 3). Therefore, prescribing these broad-spectrum antibiotics to patients will increase their risk for subsequent XDRAB-HAIs. The most likely mechanism is the selection pressure exerted by these agents on the microbial flora of the patients. In compatible with this view, we found that antibiotics with narrower spectrum, such as glycopeptide, ertapenem, and third-generation cephalosporins, are not risk factors for XDRAB-HAIs in the present study.

Our study had several limitations. First, the APACHE score for severity of illness was not measured in our study. Without this information, we were unable to explicitly show that the severity of illness is a risk factor for XDRAB-HAIs. The variable >30 days bedridden may be a proxy for severity of illness. The second limitation is the small sample size which led to a lack of power to determine the individual effects of each broad spectrum antibiotic. Finally, this was a single center study, and therefore, our findings may not be generalizable to other settings.

In conclusion, the findings of this investigation highlight that, even in the absence of clonal dissemination, XDRAB can emerge under the selection pressure of broad-spectrum antibiotics therapy, and causes subsequent HAIs in compromised hosts. An appropriate response to the continuing threat of XDRAB-HAIs should include a component of the prudent use of broad-spectrum antibiotics active against gram-negative bacteria.

## Supporting Information

Table S1 **Oligonucleotide primer sequences used for the amplification of class 1 and class 2 integrases and variable regions.**
(DOCX)
