# Supplementary material for: Risk Factors for Healthcare-Associated Extensively Drug-Resistant Acinetobacter baumannii Infections: A Case-Control Study
Source: PLoS One. 2014 Jan 21;9(1):e85973. doi: 10.1371/journal.pone.0085973 (PMC3897568; doi:10.1371/journal.pone.0085973)
Supplement: Table S1 — Oligonucleotide primer sequences used for the amplification of class 1 and class 2 integrases and variable regions. (DOCX) [file pone.0085973.s001.docx]

Table S1: Oligonucleotide primer sequences used for the amplification of class 1 and class 2 integrases and variable regions

| PCR | Primer name | Sequence | Size (bp) |
| --- | --- | --- | --- |
| Integrase | Int1 F | CAG TGG ACA TAA GCC TGT TC | 160 |
|  | Int1 R | CCC GAG GCA TAG ACT GTA |  |
|  | Int2 F | TTG CGA GTA TCC ATA ACC TG | 288 |
|  | Int2 R | TTA CCT GCA CTG GAT TAA GC |  |
| Integron | 5’CS | GGC ATC CAA GCA GCA AG |  |
|  | 3’CS | AAG CAG ACT TGA CCT GA |  |
| OXA typing | OXA-23 F | GAT CGG ATT GGA GAA CCA GA | 501 |
|  | OXA-23 R | ATT TCT GAC CGC ATT TCC AT |  |
|  | OXA-24 F | GGT TAG TTG GCC CCC TTA AA | 246 |
|  | OXA-24 R | AGT TGA GCG AAA AGG GGA TT |  |
|  | OXA-51 F | TAA TGC TTT GAT CGG CCT TG | 353 |
|  | OXA-51 R | TGG ATT GCA CTT CAT CTT GG |  |
|  | OXA-58 F | AAG TAT TGG GGC TTG TGC TG | 599 |
|  | OXA-58 R | CCC CTCTGCGCTCTACATAC |  |
